# Identification of Signal Pathways and Hub Genes of Pulmonary Arterial Hypertension by Bioinformatic Analysis

**DOI:** 10.1155/2022/1394088

**Published:** 2022-08-29

**Authors:** Rui-Qi Wei, Wen-Mei Zhang, Zhe Liang, Chunmei Piao, Guangfa Zhu

**Affiliations:** ^1^Department of Pulmonary and Critical Care Medicine, Beijing Chaoyang Hospital Affiliated to the Capital Medical University, Beijing 100020, China; ^2^Department of Pulmonary and Critical Care Medicine, Beijing Anzhen Hospital Affiliated to the Capital Medical University, Beijing 100029, China; ^3^Department of Cardiology, Peking University First Hospital, Beijing 100034, China; ^4^Beijing Anzhen Hospital Affiliated to the Capital Medical University, Beijing Institute of Heart Lung and Blood Vessel Diseases, Beijing 100029, China

## Abstract

Pulmonary arterial hypertension (PAH) is a progressive and complex pulmonary vascular disease with poor prognosis. The aim of this study was to provide a new understanding of the pathogenesis of disease and potential treatment targets for patients with PAH based on multiple-microarray analysis.Two microarray datasets (GSE53408 and GSE113439) downloaded from the Gene Expression Omnibus (GEO) database were analysed. All the raw data were processed by R, and differentially expressed genes (DEGs) were screened out by the “limma” package. Gene Ontology (GO) and Kyoto Encyclopedia of Genes and Genomes (KEGG) pathway enrichment analyses were performed and visualized by R and Cytoscape software. Protein-protein interactions (PPI) of DEGs were analysed based on the NetworkAnalyst online tool. A total of 442 upregulated DEGs and 84 downregulated DEGs were identified. GO enrichment analysis showed that these DEGs were mainly enriched in mitotic nuclear division, organelle fission, chromosome segregation, nuclear division, and sister chromatid segregation. Significant KEGG pathway enrichment included ribosome biogenesis in eukaryotes, RNA transport, proteoglycans in cancer, dilated cardiomyopathy, rheumatoid arthritis, vascular smooth muscle contraction, focal adhesion, regulation of the actin cytoskeleton, and hypertrophic cardiomyopathy. The PPI network identified 10 hub genes including HSP90AA1, CDC5L, MDM2, LRRK2, CFTR, IQGAP1, CAND1, TOP2A, DDX21, and HIF1A. We elucidated potential biomarkers and therapeutic targets for PAH by bioinformatic analysis, which provides a theoretical basis for future study.

## 1. Introduction

According to the latest clinical guidelines, pulmonary hypertension (PH) is divided into five major categories, the first of which is pulmonary arterial hypertension (PAH). PAH is a progressive and fatal disorder accompanied by nonspecific clinical manifestations such as chest pain, dyspnea, and cyanosis [1]. The main pathophysiological changes in PAH are increases in pulmonary arterial pressure and pulmonary vascular resistance (PVR) because of vascular proliferation and adverse remodeling in the distal pulmonary arteriole, giving rise to right heart failure and even death [1, 2]. Although basic discoveries and pivotal clinical trials have led to the development of medications in recent years [3], patients with PAH still have a poor prognosis. According to a previous study of Chinese people, the 1- and 3-year survival rates are only 68% and 39%, respectively, and even though survival rates for PAH have improved in China in the targeted therapy era, the mortality is still high [4]. The existing medications, including prostacyclin analogues and receptor agonists, phosphodiesterase type 5 inhibitors, and endothelin receptor antagonists, are aimed at correcting the imbalance of vasoactive factors in PAH [5, 6]. There is a shortage of new drugs to address other pathological mechanisms such as vascular remodeling [6]. Therefore, it is important to elucidate the molecular mechanisms and to identify novel drug targets for potential therapy.

Microarrays are powerful and effective tools to detect the gene expression differences between health and disease conditions. Microarray technology has been used for more than two decades and is a well-established and mature biochemical technique [7]. A large amount of microarray data is freely available from the NCBI-Gene Expression Omnibus (GEO), which is an open access database. The objective of this study was to use transcriptomic microarray data analysis to find genes associated with the onset of pulmonary hypertension, and to provide a research basis for identifying new potential therapeutic targets. In this study, we searched for and screened the transcriptome chip data of PAH from GEO to find differentially expressed genes (DEGs) between PAH and controls using bioinformatics analyses. Gene Ontology (GO) and Kyoto Encyclopedia of Genes and Genomes (KEGG) pathway enrichment analyses were performed to detect statistically significant signaling pathways of DEGs. Also, a protein-protein interaction (PPI) network was established to identify hub genes. This research will provide a direction for the discovery of diagnostic biomarkers and targeted therapy strategies related to PAH.

## 2. Materials and Methods

### 2.1. Microarray Data Source and Screening

The GEO is a public database that stores microarray, next-generation sequencing, and other high-throughput sequencing data (https://www.ncbi.nlm.nih.gov/). Using the search terms “pulmonary hypertension” and “microarray,” and after filtering by “Homo sapiens” and “Expression profiling by array,” we identified 15 transcriptomic studies. Then, only human transcriptomic studies from lung tissue and with the same GEO platform were included. We ultimately selected two GEO series (GSE) studies (GSE53408, GSE113439) for further analysis. The study information is shown in Table 1.

In the two original studies, lung tissue samples were from PAH patients undergoing lung transplantation and healthy lung tissue was derived from normal tissue adjacent to cancer during lung cancer resection. First, total RNA was extracted by the Trizol method and reverse transcribed into cDNA. Second, cRNA was obtained by in vitro transcription using cDNA as a template and labeled by biotin. Third, GeneChips were hybridized with cRNA, washed, and stained in an Affymetrix Fluidics Station 400. Finally, GeneChips were scanned using a GeneArray scanner. The two GSE studies used two different scanners.

### 2.2. Data Processing and Quality Control

GSE53408 [8] contains 11 normal and 12 severe PAH lung tissue samples, while GSE113439 [9] contains 11 normal and 15 PAH samples (one CTEPAH sample (GSM3106338) was removed because it does not belong to WHO Group 1 pulmonary hypertension). We downloaded the raw CEL files of GSE53408 and GSE113439 from the GEO database. R software (version 4.0.2) was used to process the raw data. The “affy” package [10] was used to read the CEL files and extract Affymetrix GeneChip probe level data. After correcting background, normalizing, and summarizing by using the Robust Multichip Average (RMA) algorithm [11], we got the probe expression matrix. Finally, we converted probe IDs into gene symbols (international standard names of genes) using the GPL6244 annotation package (“hugene10sttranscriptcluster.db” [12]). Probe IDs without corresponding gene symbols were deleted, and the maximum value was regarded as the gene expression value when multiple probes corresponded to one gene. We further carried out quality control for the gene expression matrix obtained above using cluster analysis and principal component analysis (PCA).

### 2.3. Screening and Integration of DEGs

We used the “limma” package [13] for DEG analysis. Gene expression values have been log2 transformed and normalized after RMA algorithm processing and were fitted to a linear model by using weighted least squares. Differential expression analysis was performed between PAH and controls. “Limma” package calculated the log2 fold change (log2FC) and false discovery rate (FDR) adjusted *P* value for each gene. FDR uses the Benjamini–Hochberg method to correct the *P* value in order to control the number of false positives for multiple tests. Adjusted *P* value <0.05 and |log2FC| ≥ 1.0 were considered as DEGs. Considering the large batch effect between GSE53408 and GSE113439, we analysed and obtained the DEGs for each dataset. Ultimate DEGs were integrated by using the Venn diagram tool (https://bioinformatics.psb.ugent.be/webtools/Venn/).

### 2.4. GO and KEGG Pathway Enrichment Analysis

GO enrichment analysis of DEGs was performed using the “enrichGO” function of the “clusterProfiler” package [14]. Genes were divided into three categories in function including biological process (BP), molecular function (MF), and cellular components (CC). GO terms were considered significantly enriched for the DEGs while an adjusted *P* value <0.05. The Benjamini and Hochberg method was used to adjust the raw *P* values. The KEGG (https://www.genome.jp/kegg/) pathway enrichment analysis of integrated DEGs in our study was performed using the DAVID webtools (https://david.ncifcrf.gov/). *P* value <0.05 was considered to be significant statistically. The visualization of analytical results was achieved by using the R (4.0.2 version) and Cytoscape software (3.8.0 version).

### 2.5. Tissue-Specific PPI Network Analysis and Hub Gene Identification

Different gene lists were inputted into NetworkAnalyst for lung tissue-specific PPI network analyses [15]. NetworkAnalyst is a bioinformatic online analysis tool with power functions and friendly operation interface, which can be accessed in https://www.networkanalyst.ca/. PPI data from human tissues were obtained from the DifferentialNet database (https://netbio.bgu.ac.il/diffnet/) [16]. Hub genes were further identified by a selected minimum connected network. Each node represents a protein encoded by a gene while connections between nodes represent the interaction of proteins. The nodes of maximum connectivity were considered as core proteins or hub genes with important biological regulatory functions in PAH.

## 3. Results

### 3.1. Microarray Data Information

**Figure 1:**
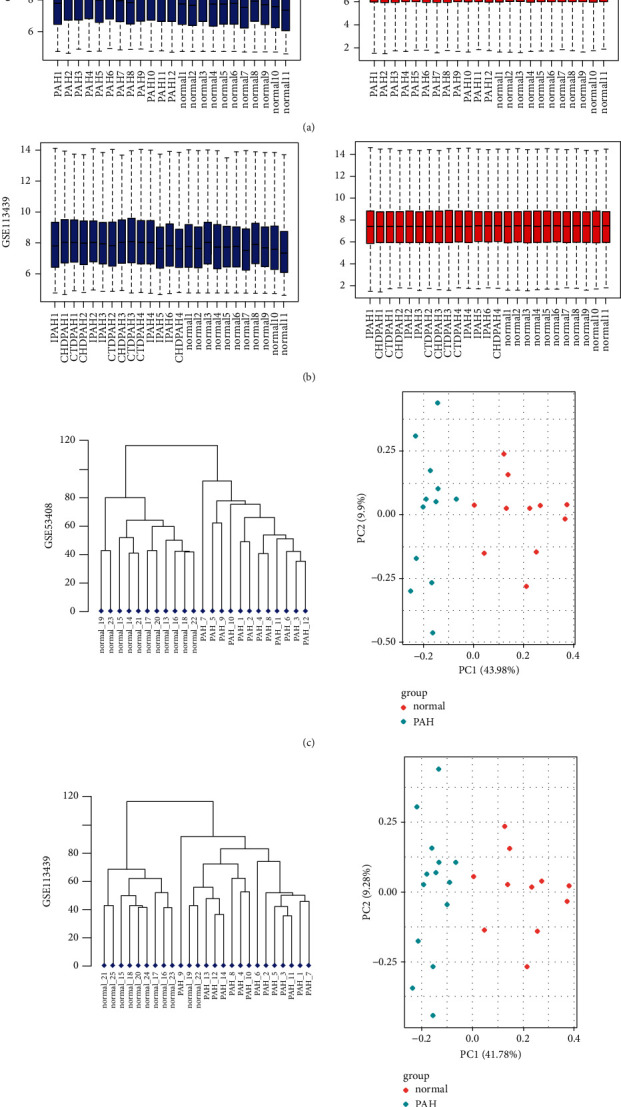
Normalization of gene expression and cluster analysis and PCA analysis for each sample. (a) Normalization of gene expression for GSE53408 data. (b) Normalization of gene expression for GSE113439 data. Abscissa shows sample lists, and ordinates show the gene expression value (log2 transformed). Blue bars represent the data before normalization, and red bars represent the normalized data. (c) Cluster analysis and PCA analysis for each sample of GSE53408 data. (d) Cluster analysis and PCA analysis for each sample of GSE113439 data. The PAH group data and the normal group data are readily distinguished. Abbreviations: IPAH, idiopathic pulmonary arterial hypertension; CTDPAH, connective tissue disease-associated PAH; CHDPAH, congenital heart disease-associated PAH.

To identify DEGs in PAH compared with healthy individuals, two gene expression profiles were selected in our study. They were GSE53408 and GSE113439. Raw data were downloaded and processed by the “affy” package. The gene expression values were normalized by the RMA integrated preprocessing algorithm. Boxplots of data before and after normalization are shown in Figure 1(a). The results of cluster analysis and PCA indicate that the PAH group and the control group data can be readily distinguished, as shown in Figure 1(b). Therefore, subsequent genic difference analysis could be carried out.

### 3.2. DEG Analysis in PAH

**Figure 2:**
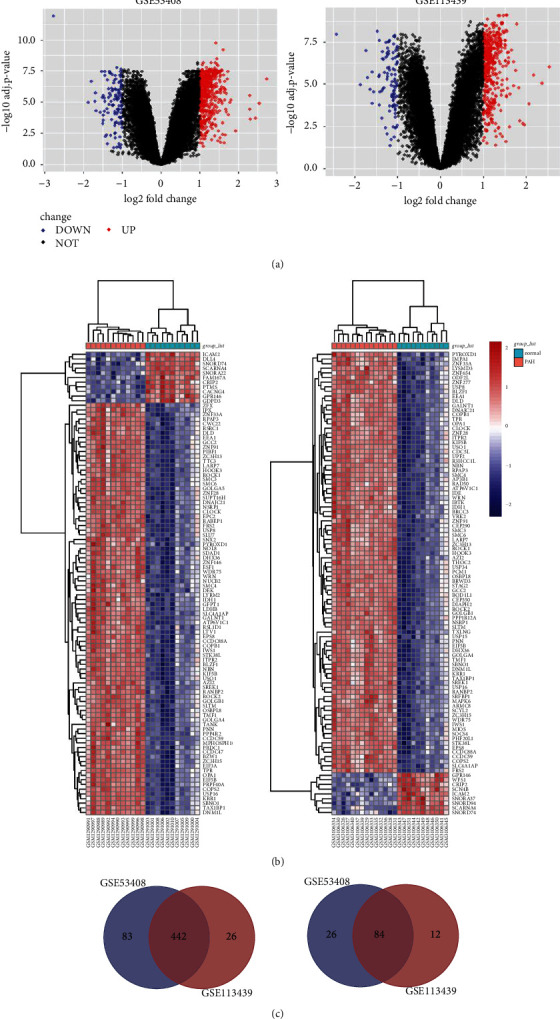
Volcano plots, cluster heatmaps, and Venn diagram of DEGs for GSE53408 and GSE113439 datasets. (a) Volcano plots of GSE53408 and GSE113439 datasets. Red dots represent upregulated genes based on adjusted *P* value <0.05 and log2FC ≥ 1. Blue dots represent downregulated genes based on adjusted *P* value<0.05 and log2FC ≤ −1. The black points represent genes with no significant difference. FC is the fold change. (b) Cluster heatmaps of the top 100 significant genes of GSE53408 and GSE113439 data. Red indicates that the gene expression is upregulated, blue indicates that the gene expression is downregulated, and white indicates no significant changes in gene expression. Abbreviations: DEGs, differentially expressed genes. (c) Venn diagram of DEGs, left panel: the upregulated overlapping DEGs in the two datasets are 442; right panel: the downregulated overlapping DEGs in the two datasets are 84.

DEGs between PAH and normal lung tissues were evaluated by the “limma” package. There were 635 DEGs in GSE53408, including 525 upregulated genes and 110 downregulated genes (Figure 2(a)). And 564 DEGs were screened in GSE113439, including 468 upregulated genes and 96 downregulated genes (Figure 2(a)). And the top 100 DEGs are displayed in the form of heatmaps, as shown in Figure 2(b). Taking into account the original experimental processes such as different model Genechip scanners, the two sets of data have a large batch effect, so a Venn diagram was constructed to obtain the final DEGs, as shown in Figure 2(c). Finally, 526 DEGs were identified in total, with 442 upregulated genes and 84 downregulated genes (Table 2). The top twenty upregulated and downregulated DEGs sorted by average log2FC size of the two datasets are shown in Table S1.

### 3.3. GO Term Enrichment Analysis of DEGs

**Figure 3:**
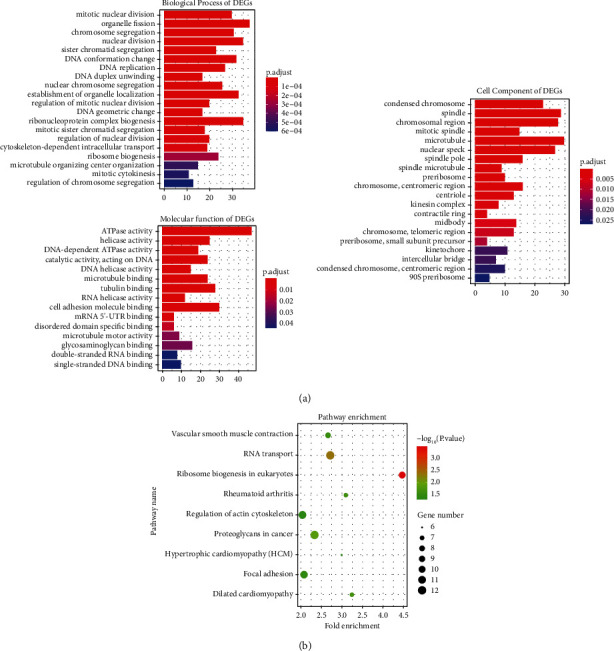
Barplots of GO enrichment analysis (top 20) and Dotplot of KEGG pathway enrichment analysis. (a) *Y*-axes show GO enrichment significance items of DEGs in three different functional groups (molecular function, biological processes, and cell composition), and *x*-axes represent the gene counts enriched in each GO term. Different bar colors represent different adjust *P* values. Abbreviations: DEGs, differentially expressed genes; GO, gene ontology. (b) Dotplot of KEGG pathway enrichment analysis. Each dot represents a KEGG pathway, and the size of each dot indicates the number of genes in the enrichment pathway.

GO enrichment analysis for integrated DEGs was performed using the “clusterProfiler” package. The enriched GO terms were divided into three ontologies including biological process (BP), molecular function (MF), and cell component (CC). Significant results of GO enrichment analysis are exhibited in Figure 3(a). In BP, DEGs were generally enriched in mitotic nuclear division, organelle fission, chromosome segregation, nuclear division, and sister chromatid segregation. In MF, DEGs were chiefly enriched in ATPase activity, helicase activity, DNA-dependent ATPase activity, catalytic activity acting on DNA, and DNA helicase activity. In CC, DEGs were mainly enriched in condensed chromosome, spindle, chromosomal region, mitotic spindle, and microtubule (Table S2 and Figure S1).

### 3.4. KEGG Pathway Enrichment Analysis of DEGs

**Figure 4:**
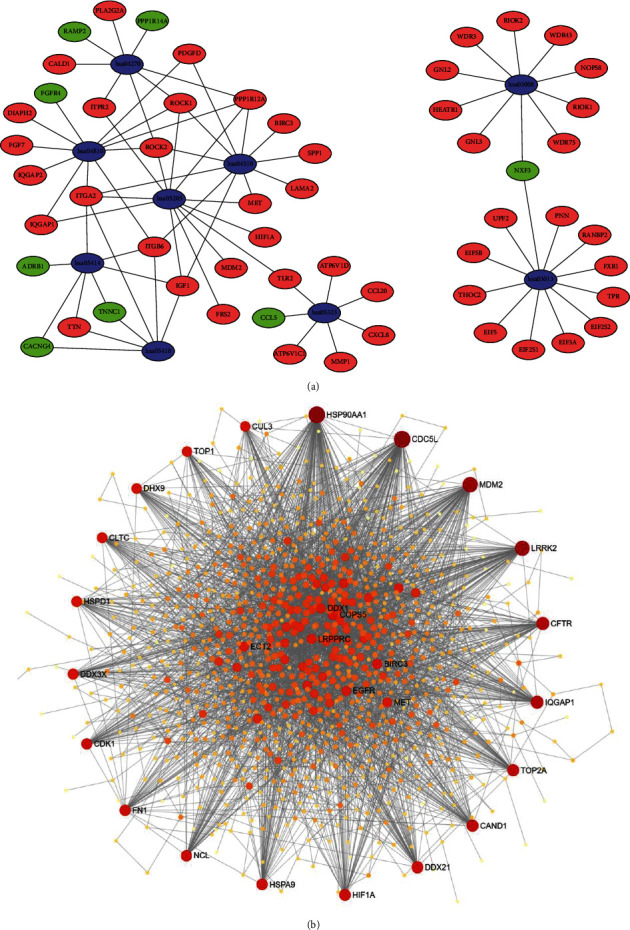
Significant pathway enrichment of DEGs and PPI network analyses of DEGs in lung tissues of PAH patients. (a) Significant pathway enrichment of DEGs, blue represents the signaling pathway, green represents downregulated genes, and red represents upregulated genes. (b) PPI network analyses of DEGs in lung tissues of PAH patients. DEGs are shown in orange. Most significant hub genes according to the highest number of connections, are arranged in the outermost circle.

After inputting the DEGs list into DAVID online tool, the most significantly enriched KEGG pathways of the DEGs were obtained (Table S3). The signaling pathways of DEGs were eventually enriched in ribosome biogenesis in eukaryotes, RNA transport, proteoglycans in cancer, dilated cardiomyopathy, rheumatoid arthritis, vascular smooth muscle contraction, focal adhesion, regulation of actin cytoskeleton, and hypertrophic cardiomyopathy (Figure 3(b)). Cytoscape software was used to calculate the network topological characteristics and draw each node and edge. Different pathway terms and genes are displayed by ovals in different colors, as shown in Figure 4(a).

### 3.5. Tissue-Specific PPI Network Analysis and Hub Gene Identification

Protein-protein interactions among the DEGs were predicted by the NetworkAnalyst tool. Human lung tissue-specific networks were constructed based on the DifferentialNet database. We chose a minimum connected network for identifying hub genes in PAH. This PPI network contains 1030 nodes and 3364 edges (Figure 4(b)). The top ten hub genes evaluated according to the degree of connectivity in the PPI network were HSP90AA1, CDC5L, MDM2, LRRK2, CFTR, IQGAP1, CAND1, TOP2A, DDX21, and HIF1A (Table 3). All of these identified hub genes were upregulated in PAH patients.

## 4. Discussion

Pulmonary arterial hypertension is a chronic and progressive disease with few typical manifestations and thus could be delayed in diagnosis and treatment [2]. Unfortunately, the fundamental pathogenesis of PAH preliminarily remains unclear warranting the necessity to explore basic mechanisms to delineate specific targets promising for therapeutic intervention.

In this study, we downloaded two microarray datasets, GSE53408 and GSE113439, from the GEO database and separately obtained the DEGs by bioinformatics analysis. Then the upregulated and downregulated genes from two datasets were intersected by a Venn diagram webtool to obtain the final integrated DEGs. We identified 526 DEGs, including 442 upregulated genes and 84 downregulated genes. The integrated DEGs were further subjected to GO and KEGG pathway enrichment analysis and tissue-specific PPI network analysis. There has been a number of PAH studies using microarray analyses [17–19], and the signaling pathway or hub genes identified by each study varied widely. This was mainly due to different microarray platforms or different data processing procedures. In Li's study [18], they analysed the DEGs using the GEO2R online tool. Although the GEO2R tool is simple and convenient, the average gene expression value varies from one sample to another in the raw data, so it is hard to ensure the accuracy of the analysis results. Our study started by analyzing raw CEL format microarray data and normalized it using the RMA algorithm to keep the gene expression levels of each sample at the same level, which ensured the reliability of downstream analysis. Luo et al. [17] combined the disease group and control samples of the two datasets and used the “sva” R package to remove batch effects for DEG analysis that may cause some deviation considering the use of different GeneArray scanners between GSE53408 and GSE113439. In this study, we analysed GSE53408 and GSE113439 and merged the DEGs of the two datasets, which may be more reasonable. In addition, one CTEPH sample in GSE113439 was not selected in our study because we only paid attention to the WHO group 1 PH.

The DEGs associated with GO BP terms mainly consisted of mitotic nuclear division, organelle fission, chromosome segregation, nuclear division, and sister chromatid segregation, which all participated in the process of cell division and proliferation. In PAH progression, excessive proliferation of pulmonary artery smooth muscle cells plays an important role, which causes vascular medial thickening and pulmonary vascular remodeling [20]. DEGs associated with GO MF terms such as ATPase activity, helicase activity, DNA-dependent ATPase activity, catalytic activity acting on DNA, and DNA helicase activity indicated that DNA replication was active, which was consistent with the results of BP. The enriched KEGG pathways of DEGs such as vascular smooth muscle contraction, focal adhesion, and regulation of the actin cytoskeleton may be associated with the progression of PAH. As we all know, pulmonary vascular smooth muscle contraction is a critical pathological mechanism of PAH, especially in the state induced by hypoxia. Hypoxia promoted pulmonary vasoconstriction by interacting with a variety of protein kinases, calcium channels, and potassium channels [21]. Blocking these signal transduction pathways may provide novel therapeutic approaches for PAH depending on further research. The focal adhesion pathway was also identified in PAH by Zhao and Austin's study [22]. Focal adhesion dynamics is regulated by focal adhesion kinase (FAK) family signaling via integrins [23]. Several studies have demonstrated the association of FAK with vascular diseases including PAH [23, 24]. Jia et al. [24] found that activation of FAK could stimulate pulmonary arterial smooth muscle cell proliferation in vitro. Paulin et al. [25] confirmed that FAK inhibitor improved hemodynamics, vascular remodeling, and right ventricular hypertrophy in monocrotaline induced PAH rat model. Based on the above research, FAK inhibition may open a new therapeutic way for PAH. Finally, regulation of the actin cytoskeleton was found to be involved in the process of smooth muscle cell contraction, proliferation, and migration [26]. Abelson tyrosine kinase (c-Abl) could modulate restructuring of actin cytoskeletal [27]. There was some research [28, 29] suggesting that c-Abl inhibitor imatinib attenuated the symptoms of patients with PAH.

Network analysis is a powerful tool that helps to identify hub genes and to discover novel targets for therapy [30]. We constructed a PPI network of proteins encoded by DEGs and screened 10 hub genes according to their connectivity degree: HSP90AA1, CDC5L, MDM2, LRRK2, CFTR, IQGAP1, CAND1, TOP2A, DDX21, and HIF1A. We searched for the relationship between each gene symbol and pulmonary hypertension in PubMed and found that four genes involved in PAH had been studied by previous researchers. Shen et al. [31] reported that MDM2-mediated ubiquitination of ACE2 contributed to the development of PAH, and the MDM2 inhibitor JNJ-165 reversed SU5416/hypoxia-induced PAH in mice model. CFTR is expressed in PASMCs and modulates pulmonary arterial tone [32]. In hypoxia-induced PAH mice, deficiency of CFTR signally attenuated right ventricular systolic pressure, pulmonary vessel medial wall thickness, and muscularization [33]. Grigolo et al. [34] found that antitopoisomerase II (TOP2A) autoantibodies were related to systemic sclerosis associated with pulmonary hypertension. Luo et al. [35] reported that the CD146-HIF-1*α* axis in PASMC derived vascular remodeling and PAH. Modulation of CD146-HIF-1*α* may be a new antiremodeling therapy strategy. The remaining six hub genes, HSP90AA1, CDC5L, LRRK2, IQGAP1, CAND1, and DDX21, were not found to have a direct connection with PAH previously. HSP90AA1, as a molecular chaperone, assisted the assembly of intracellular molecules and protein folding, and participated in multiple processes of fibroblast activation [36, 37]. Upregulation of CDC5L could promote cell cycle progression and proliferation [38]. Hongge et al. [39] found that LRRK2 was expressed in endothelial cells and regulated the procedure of monocyte adhesion, which was involved in vascular inflammation and immune response. IQGAP1, as a scaffold protein, maintained the integrity of the endothelium barrier [40]. Gharib et al. [41] discovered that IQGAP1 was a novel candidate biomarker and played a crucial role in hypoxia-induced pulmonary hypertension and vascular remodeling by global gene annotation analysis.

There are several limitations in our study. At first, microarray technology can only detect known genes, and due to discrepancies in chip probes designed by different companies, the conclusions obtained above might vary. Then, considering the relatively small sample size in the two public datasets (48 samples in total), there may be some bias on screened DEGs. Finally, all of our results were based on the forecasting of bioinformatic analysis, which should be further confirmed by *in vitro* and *in vivo* experiments.

## 5. Conclusion

In summary, we analysed the DEGs between patients and healthy individuals aiming to assist the understanding of the pathogenesis of PAH. Vascular smooth muscle contraction, focal adhesion, and regulation of the actin cytoskeleton pathways were found to be involved in PAH in this study. And ten hub genes, including HSP90AA1, CDC5L, MDM2, LRRK2, CFTR, IQGAP1, CAND1, TOP2A, DDX21, and HIF1A may be new therapeutic targets for patients with PAH to be further validated.

## Figures and Tables

**Table 1:** Details for PAH microarray study.

Reference	Sample	Dataset	Platform	Normal	PAH
Zhao et al. [8]	Lung tissue	GSE53408	GPL6244	11	12
Mura et al. [9]	Lung tissue	GSE113439	GPL6244	11	14

Abbreviations: PAH, pulmonary arterial hypertension.

**Table 2:** Number of differentially expressed genes.

GSE datasets	Upregulated	Downregulated	Total
GSE53408	525	110	635
GSE113439	468	96	564
Overlapped genes	442	84	526

**Table 3:** Top 10 hub genes.

Hub gene	Description	Degree
HSP90AA1	Heat shock protein 90 alpha family class a member 1	121
CDC5L	Cell division cycle 5 like	114
MDM2	MDM2 proto-oncogene	94
LRRK2	Leucine rich repeat kinase 2	88
CFTR	Cystic fibrosis transmembrane conductance regulator	60
IQGAP1	IQ motif containing GTPase activating protein 1	59
CAND1	Cullin associated and neddylation dissociated 1	48
TOP2A	DNA topoisomerase II alpha	48
DDX21	DExD-box helicase 21	46
HIF1A	Hypoxia inducible factor 1 subunit alpha	45

## Data Availability

All data relevant to the study are included in the article and are available from the corresponding author on reasonable request.

## Supplementary Materials

Supplementary Materials Figure S1: genes enriched in each GO term. (a) Biological process of DEGs; (b) molecular function of DEGs; (c) cell component of DEGs. Brown dots represent GO terms, and black dots represent genes. Table S1: top 20 upregulated and downregulated DEGs according to average gene expression fold change from two datasets. Table S2: GO enriched analysis of DEGs between PAH and normal tissues (top five terms in BP, MF, and CC of DEGs according to *P* value). Table S3: KEGG enriched analysis of DEGs between PAH and normal tissues.

## References

[1] Humbert M., Guignabert C., Bonnet S. (2019). Pathology and pathobiology of pulmonary hypertension: state of the art and research perspectives. *European Respiratory Journal*.

[2] Kim D., George M. P. (2019). Pulmonary hypertension. *Medical Clinics of North America*.

[3] Klinger J. R., Elliott C. G., Levine D. J. (2019). Therapy for pulmonary arterial hypertension in adults: update of the chest guideline and expert panel report. *Chest*.

[4] Zhang R., Dai L. Z., Xie W. P. (2011). Survival of Chinese patients with pulmonary arterial hypertension in the modern treatment era. *Chest*.

[5] Galie N., Humbert M., Vachiery J. L. (2015). Esc/ers guidelines for the diagnosis and treatment of pulmonary hypertension: the joint task force for the diagnosis and treatment of pulmonary hypertension of the European society of cardiology (esc) and the European respiratory society (ers): endorsed by: association for European paediatric and congenital cardiology (aepc), international society for heart and lung transplantation (ishlt). *European Heart Journal*.

[6] Sitbon O., Gomberg-Maitland M., Granton J. (2019). Clinical trial design and new therapies for pulmonary arterial hypertension. *European Respiratory Journal*.

[7] Hoffmann J., Wilhelm J., Olschewski A., Kwapiszewska G. (2016). Microarray analysis in pulmonary hypertension. *European Respiratory Journal*.

[8] Zhao Y. D., Yun H. Z. H., Peng J. (2014). De novo synthesize of bile acids in pulmonary arterial hypertension lung. *Metabolomics*.

[9] Mura M., Cecchini M. J., Joseph M., Granton J. T. (2019). Osteopontin lung gene expression is a marker of disease severity in pulmonary arterial hypertension. *Respirology*.

[10] Gautier L., Cope L., Bolstad B. M., Irizarry R. A. (2004). Affy--Analysis of Affymetrix Genechip data at the probe level. *Bioinformatics*.

[11] Irizarry R. A., Hobbs B., Collin F. (2003). Exploration, normalization, and summaries of high density oligonucleotide array probe level data. *Biostatistics*.

[12] MacDonald J. W. (2017). Hugene10sttranscriptcluster.Db: Affymetrix Hugene10 annotation data (chip Hugene10sttranscriptcluster). https://bioconductor.org/packages/release/data/annotation/html/hugene10sttranscriptcluster.db.html.

[13] Ritchie M. E., Phipson B., Wu D. (2015). Limma powers differential expression analyses for rna-sequencing and microarray studies. *Nucleic Acids Research*.

[14] Yu G., Wang L. G., Han Y., He Q. Y. (2012). Clusterprofiler: an R package for comparing biological themes among gene clusters. *OMICS: A Journal of Integrative Biology*.

[15] Xia J., Benner M. J., Hancock R. E. (2014). Networkanalyst—Integrative approaches for protein-protein interaction network analysis and visual exploration. *Nucleic Acids Research*.

[16] Basha O., Shpringer R., Argov C. M., Yeger-Lotem E. (2018). The differentialnet database of differential protein-protein interactions in human tissues. *Nucleic Acids Research*.

[17] Luo J., Li H., Liu Z. (2020). Integrative analyses of gene expression profile reveal potential crucial roles of mitotic cell cycle and microtubule cytoskeleton in pulmonary artery hypertension. *BMC Medical Genomics*.

[18] Li Q., Meng L., Liu D. (2020). Screening and identification of therapeutic targets for pulmonary arterial hypertension through microarray technology. *Frontiers in Genetics*.

[19] Zhao E., Xie H., Zhang Y. (2021). Identification of differentially expressed genes associated with idiopathic pulmonary arterial hypertension by integrated bioinformatics approaches. *Journal of Computational Biology*.

[20] Morii C., Tanaka H. Y., Izushi Y. (2020). 3d in vitro model of vascular medial thickening in pulmonary arterial hypertension. *Frontiers in Bioengineering and Biotechnology*.

[21] Sommer N., Strielkov I., Pak O., Weissmann N. (2016). Oxygen sensing and signal transduction in hypoxic pulmonary vasoconstriction. *European Respiratory Journal*.

[22] Zhao M., Austin E. D., Hemnes A. R., Loyd J. E., Zhao Z. (2014). An evidence-based knowledgebase of pulmonary arterial hypertension to identify genes and pathways relevant to pathogenesis. *Molecular BioSystems*.

[23] Murphy J. M., Jeong K., Lim S. T. S. (2020). Fak family kinases in vascular diseases. *International Journal of Molecular Sciences*.

[24] Jia D., Zhu Q., Liu H. (2017). Osteoprotegerin disruption attenuates HySu-induced pulmonary hypertension through integrin *α* v *β*_3_/FAK/AKT pathway suppression. *Circulation: Cardiovascular Genetics*.

[25] Paulin R., Meloche J., Courboulin A. (2014). Targeting cell motility in pulmonary arterial hypertension. *European Respiratory Journal*.

[26] Tang D. D. (2018). The dynamic actin cytoskeleton in smooth muscle. *Advances in Pharmacology*.

[27] Cleary R. A., Wang R., Wang T., Tang D. D. (2013). Role of abl in airway hyperresponsiveness and airway remodeling. *Respiratory Research*.

[28] Patterson K. C., Weissmann A., Ahmadi T., Farber H. W. (2006). Imatinib mesylate in the treatment of refractory idiopathic pulmonary arterial hypertension. *Annals of Internal Medicine*.

[29] Ghofrani H. A., Seeger W., Grimminger F. (2005). Imatinib for the treatment of pulmonary arterial hypertension. *New England Journal of Medicine*.

[30] Ozturk K., Dow M., Carlin D. E., Bejar R., Carter H. (2018). The emerging potential for network analysis to inform precision cancer medicine. *Journal of Molecular Biology*.

[31] Shen H., Zhang J., Wang C. (2020). Mdm2-Mediated ubiquitination of angiotensin-converting enzyme 2 contributes to the development of pulmonary arterial hypertension. *Circulation*.

[32] Robert R., Savineau J. P., Norez C., Becq F., Guibert C. (2007). Expression and function of cystic fibrosis transmembrane conductance regulator in rat intrapulmonary arteries. *European Respiratory Journal*.

[33] Tabeling C., Yu H., Wang L. (2015). Cftr and sphingolipids mediate hypoxic pulmonary vasoconstriction. *Proceedings of the National Academy of Sciences of the U S A*.

[34] Grigolo B., Mazzetti I., Meliconi R. (2001). Anti-topoisomerase ii alpha autoantibodies in systemic sclerosis-association with pulmonary hypertension and hla-B35. *Clinical and Experimental Immunology*.

[35] Luo Y., Teng X., Zhang L. (2019). CD146-HIF-1*α* hypoxic reprogramming drives vascular remodeling and pulmonary arterial hypertension. *Nature Communications*.

[36] Sontake V., Wang Y., Kasam R. K. (2017). Hsp90 regulation of fibroblast activation in pulmonary fibrosis. *JCI Insight*.

[37] Bellaye P. S., Shimbori C., Yanagihara T. (2018). Synergistic role of HSP90*α* and HSP90*β* to promote myofibroblast persistence in lung fibrosis. *European Respiratory Journal*.

[38] Liu H., Guo D., Sha Y. (2020). Anxa7 promotes the cell cycle, proliferation and cell adhesion-mediated drug resistance of multiple myeloma cells by up-regulating Cdc5l. *Aging (Albany NY)*.

[39] Hongge L., Kexin G., Xiaojie M., Nian X., Jinsha H. (2015). The role of Lrrk2 in the regulation of monocyte adhesion to endothelial cells. *Journal of Molecular Neuroscience*.

[40] Mehta D., Ravindran K., Kuebler W. M. (2014). Novel regulators of endothelial barrier function. *American Journal of Physiology—Lung Cellular and Molecular Physiology*.

[41] Gharib S. A., Luchtel D. L., Madtes D. K., Glenny R. W. (2005). Global gene annotation analysis and transcriptional profiling identify key biological modules in hypoxic pulmonary hypertension. *Physiological Genomics*.

