# Supplementary material for: Identification of Signal Pathways and Hub Genes of Pulmonary Arterial Hypertension by Bioinformatic Analysis
Source: Can Respir J. 2022 Aug 29;2022:1394088. doi: 10.1155/2022/1394088 (PMC9444450; doi:10.1155/2022/1394088)
Supplement: Supplementary Materials — Figure S1: genes enriched in each GO term. (a) Biological process of DEGs; (b) molecular function of DEGs; (c) cell component of DEGs. Brown dots represent GO terms, and black dots represent genes. Table S1: top 20 upregulated and downregulated DEGs according to average gene expression fold change from two datasets. Table S2: GO enriched analysis of DEGs between PAH and normal tissues (top five terms in BP, MF, and CC of DEGs according to P value). Table S3: KEGG enriched analysis of DEGs between PAH and normal tissues. [file 1394088.f1.zip › Supplementary table.docx]

**TABLE S1: Top 20 up- and down-regulated DEGs according to average gene expression fold change from two datasets**

| DEGs | Gene symbol |
| --- | --- |
| Upregulated | POSTN SLC7A11 TDO2 PI15 VSIG1 SPP1 MMP8 TTN SNORD20 SMC4 ANLN HSPH1 CD163 DNTTIP2 CFH ANGPT2 HSPD1 ZNF267 TOP2A HSP90AA1 |
| Downregulated | SCARNA4 SOSTDC1 RAMP2 S100A3 MS4A15 HIGD1B RNU5D-1 MYOZ1 VIPR1 VTRNA1-1 GPR146 HEY1 GDPD3 SNORD94 TMEM130 TMEM100 NKG7 ECRG4 ST8SIA6 DEPP1 |

**TABLE S2: GO enriched analysis of DEGs between PAH and normal tissues (top five terms in BP, MF, and CC of DEGs according to P value)**

| **Term** | **Description** | **Gene count** | **Category** | **P value** |
| --- | --- | --- | --- | --- |
| GO:0140014 | mitotic nuclear division | 30 | BP | 1.45E-11 |
| GO:0048285 | organelle fission | 38 | BP | 2.08E-10 |
| GO:0007059 | chromosome segregation | 31 | BP | 4.19E-10 |
| GO:0000280 | nuclear division | 35 | BP | 7.22E-10 |
| GO:0000819 | sister chromatid segregation | 23 | BP | 9.34E-10 |
| GO:0016887 | ATPase activity | 47 | MF | 3.53E-16 |
| GO:0004386 | helicase activity | 25 | MF | 1.79E-12 |
| GO:0008094 | DNA-dependent ATPase activity | 19 | MF | 1.67E-10 |
| GO:0140097 | catalytic activity, acting on DNA | 24 | MF | 3.36E-09 |
| GO:0003678 | DNA helicase activity | 15 | MF | 3.58E-09 |
| GO:0000793 | condensed chromosome | 23 | CC | 1.55E-08 |
| GO:0005819 | spindle | 29 | CC | 2.54E-08 |
| GO:0098687 | chromosomal region | 28 | CC | 9.29E-08 |
| GO:0072686 | mitotic spindle | 15 | CC | 1.18E-07 |
| GO:0005874 | microtubule | 30 | CC | 3.74E-07 |

**Notes:** BP, biological process; MF, molecular function; CC, cell component.

**TABLE S3: KEGG enriched analysis of DEGs between PAH and normal tissues**

| **Category** | **Term** | **Description** | **Gene count** | **P value** |
| --- | --- | --- | --- | --- |
| KEGG pathway | hsa03008 | Ribosome biogenesis in eukaryotes | 10 | 3.53E-04 |
| KEGG pathway | hsa03013 | RNA transport | 12 | 4.48E-03 |
| KEGG pathway | hsa05205 | Proteoglycans in cancer | 12 | 1.33E-02 |
| KEGG pathway | hsa05414 | Dilated cardiomyopathy | 7 | 2.02E-02 |
| KEGG pathway | hsa05323 | Rheumatoid arthritis | 7 | 2.48E-02 |
| KEGG pathway | hsa04270 | Vascular smooth muscle contraction | 8 | 3.00E-02 |
| KEGG pathway | hsa04510 | Focal adhesion | 11 | 3.82E-02 |
| KEGG pathway | hsa04810 | Regulation of actin cytoskeleton | 11 | 4.26E-02 |
| KEGG pathway | hsa05410 | Hypertrophic cardiomyopathy | 6 | 4.89E-02 |
