# Supplementary figures and images for: Identification of Signal Pathways and Hub Genes of Pulmonary Arterial Hypertension by Bioinformatic Analysis
Source: Can Respir J. 2022 Aug 29;2022:1394088. doi: 10.1155/2022/1394088 (PMC9444450; doi:10.1155/2022/1394088)

## Slide 1
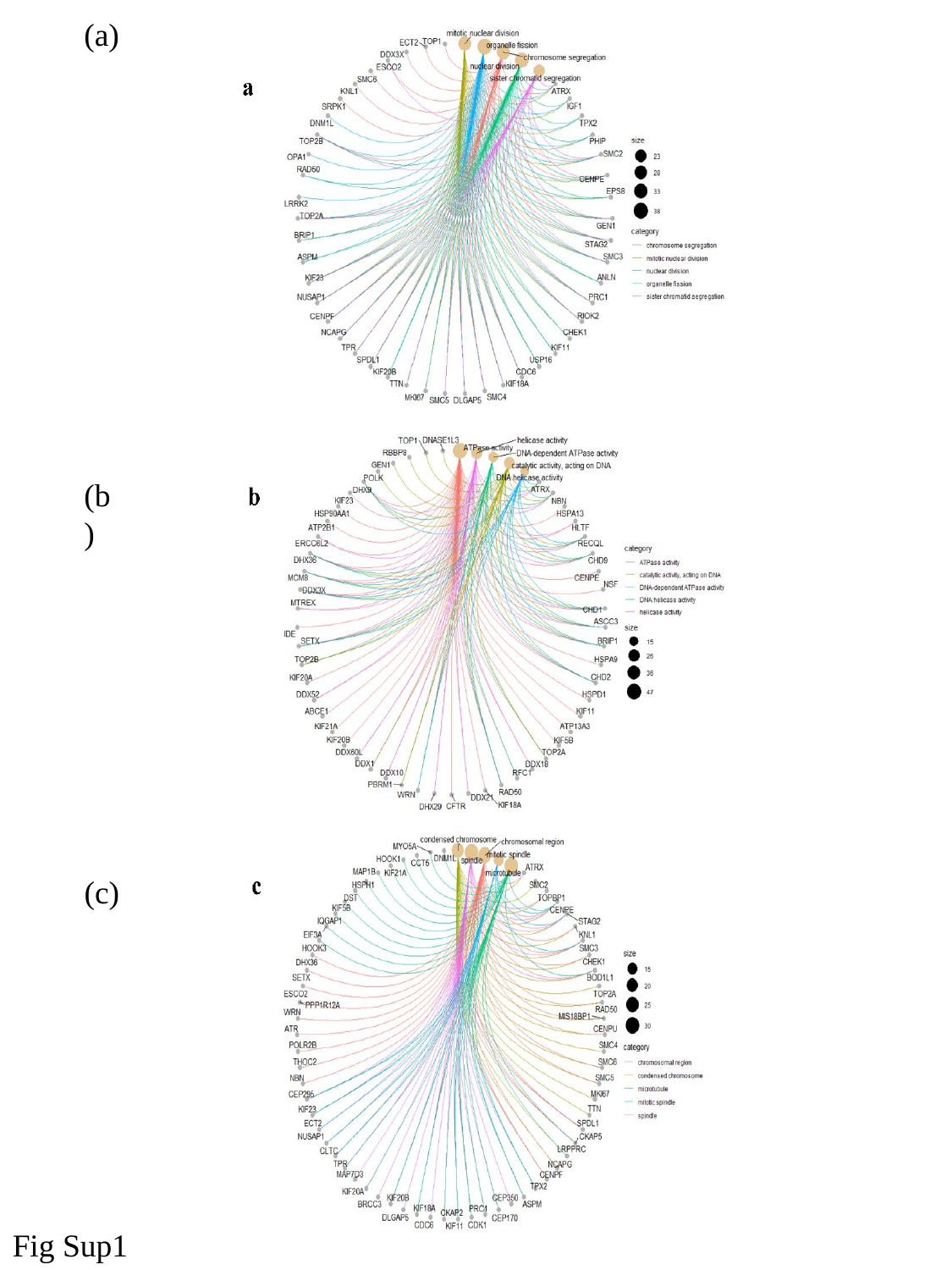

(a)
(b)
(c)
Fig Sup1

Supplement: Supplementary Materials — Figure S1: genes enriched in each GO term. (a) Biological process of DEGs; (b) molecular function of DEGs; (c) cell component of DEGs. Brown dots represent GO terms, and black dots represent genes. Table S1: top 20 upregulated and downregulated DEGs according to average gene expression fold change from two datasets. Table S2: GO enriched analysis of DEGs between PAH and normal tissues (top five terms in BP, MF, and CC of DEGs according to P value). Table S3: KEGG enriched analysis of DEGs between PAH and normal tissues. [file 1394088.f1.zip › Supp FIG.pptx]
